# Assessment of Basic Motions and Technique Identification in Classical Cross-Country Skiing

**DOI:** 10.3389/fpsyg.2019.01260

**Published:** 2019-06-07

**Authors:** Johannes Tjønnås, Trine M. Seeberg, Ole Marius Hoel Rindal, Pål Haugnes, Øyvind Sandbakk

**Affiliations:** ^1^Sintef Digital, Trondheim, Norway; ^2^Department of Neuromedicine and Movement Science, Centre for Elite Sports Research, Norwegian University of Science and Technology, Trondheim, Norway

**Keywords:** cross-country skiing, inertial measurement unit (IMU), motion estimators, technique definition, technique classification

## Abstract

Cross-country skiing is a popular Olympic winter sport, which is also used extensively as a recreational activity. While cross-country skiing primarily is regarded as a demanding endurance activity it is also technically challenging, as it contains two main styles (classical and skating) and many sub-techniques within these styles. To further understand the physiological demands and technical challenges of cross-country skiing it is imperative to identify sub-techniques and basic motion features during training and competitions. Therefore, this paper presents features for identification and assessment of the basic motion patterns used during classical-style cross-country skiing. The main motivation for this work is to contribute to the development of a more detailed platform for comparing and communicating results from technique analysis methods, to prevent unambiguous definitions and to allow more precise discussions and quality assessments of an athlete's technical ability. To achieve this, our paper proposes formal motion components and classical style technique definitions as well as sub-technique classifiers. This structure is general and can be used directly for other cyclic activities with clearly defined and distinguishable sub-techniques, such as the skating style in cross country skiing. The motion component features suggested in our approach are arm synchronization, leg kick, leg kick direction, leg kick rotation, foot/ski orientation and energy like measures of the arm, and leg motion. By direct measurement, estimation, and the combination of these components, the traditional sub-techniques of diagonal stride, double poling, double poling kick, herringbone, as well as turning techniques can be identified. By assuming that the proposed definitions of the classical XC skiing sub-techniques are accepted, the presented classifier is proven to map measures from the motion component definitions to a unique representation of the sub-techniques. This formalization and structure may be used on new motion components, measurement principles, and classifiers, and therefore provides a framework for comparing different methodologies. Pilot data from a group of high-level cross-country skiers employing inertial measurement sensors placed on the athlete's arms and skis are used to demonstrate the approach. The results show how detailed sub-technique information can be coupled with physical, track, and environmental data to analyze the effects of specific motion patterns, to develop useful debriefing tools for coaches and athletes in training and competition settings, and to explore new research hypotheses.

## 1. Introduction

Cross-country (XC) skiing is a popular Olympic winter sport but is also used extensively as a recreational activity. XC skiing is regarded as one of the most demanding of endurance sports and involves competitions on varying terrain where skiers employ different sub-techniques of the classical and skating styles. These sub-techniques include different technical features that require upper- and/or lower-body work to different extents. Specifically, XC skiers are continuously changing between and adapting the sub-techniques of the classical style—diagonal stride (DIA), double poling (DP), double poling with a kick (DK), and herringbone (HRB)—and skating style—paddle dance (G2), double dance (G3), single dance (G4), and skating without poles (G5)—to the varying terrain. In addition, the downhill tuck position (TCK) and a variety of turn techniques (TRN) are employed with both styles. Accordingly, XC skiers design their training to improve not only their physiological capacities, but also their technical and tactical expertise (Smith, 1992; Nilsson et al., 2004; Andersson et al., 2010; Bolger et al., 2015; Sandbakk and Holmberg, 2017).

The skiers' continuous choice of sub-technique is influenced by speed and external conditions (e.g., the profile of the track, snow conditions, waxing of skis, etc.), as well as individual performance level and physical characteristics. Thus, skiers must choose the terrain they train in and thereby the extent to which they train the different sub-techniques and technical features purposefully. In the classical style, DIA is primarily used in moderately steep to steep terrain, with the arms and legs moving in a manner similar to that of walking (Pellegrini et al., 2013; Dahl et al., 2017). During DP, propulsive forces are generated solely with the poles by the symmetrical and synchronous movement of both arms supported by considerable trunk flexion while crossing relatively flat terrain (Holmberg et al., 2005; Danielsen et al., 2015). In DK the upper-body movement is quite similar to the movement in DP but with additional propulsion from a left or right DIA-like leg kick. DK is normally used while traversing slightly uphill and flat terrain, depending on resistance imposed by the snow conditions (Lindinger et al., 2009a). The HRB is used during very steep uphill runs (Andersson et al., 2014b), whereas the TCK is used during downhill runs, and various TRN are employed during turns and track changes (Bucher Sandbakk et al., 2014).

In addition to mastering the different sub-techniques, a skier needs to adapt and efficiently shift between these, at speeds ranging from 5 to 70 km/h on inclines ranging from –20 to +20 percent gradients (Sandbakk and Holmberg, 2014). In the classical techniques, attaining higher speed requires both the production of sufficient propulsive force to increase cycle length, as well as more rapid cycles. Longer cycles are particularly important at high speeds on flat terrain, whereas rapid shorter cycles are mandatory for accelerating on steep hills, and during the start and sprinting at the finish of races (Lindinger et al., 2009b; Stöggl and Müller, 2009; Zory et al., 2009; Mikkola et al., 2013; Andersson et al., 2014b; Haugnes et al., 2018). To accomplish this, more explosive techniques, such as “running diagonal” and “kangaroo” double poling, have been developed (Holmberg et al., 2005; Andersson et al., 2014a). In addition, there is increasing focus on the downhill sections of a race, especially the challenging downhill turns, where faster skiers utilize the accelerating step-turn technique more extensively (Sandbakk et al., 2014). In order to properly communicate and study the effects of such motion patterns, more formal, detailed, and nuanced descriptions of the sub-techniques are needed. Such understanding contributes by providing a communication platform of high relevance for researchers in this field, as well as for developing preparation and debriefing tools for coaches and athletes in the new age of digital coaching.

Describing the motion patterns during training and competitions is now possible due to micro-sensor technology, which has revolutionized the possibilities of performing advanced field analyses of XC skiing. Since further understanding of both the physiological demands and technical challenges depends on detecting the sub-techniques, it is imperative to identify the features needed to classify sub-techniques and to describe the motion quality. The field of automatic identification of sub-techniques within XC skiing styles is getting more mature, and various models utilizing different methodologies are being published with increasing precision and accuracy of the classification results (Myklebust et al., 2011, 2015; Marsland et al., 2012, 2015, 2017, 2018; Holst and Jonasson, 2013; Sakurai et al., 2014, 2016; Stöggl et al., 2014; Rindal et al., 2017; Seeberg et al., 2017; Jang et al., 2018; Solli et al., 2018). However, comparing precision and specific results across different studies is challenging due to the lack of detailed quantitative sub-technique definitions. In addition, the results from different studies depend on the sensor system and placement of sensors, the experimental setup and protocol, the methodology for classification, the athlete's capability and instructions on how to ski, as well as the expert labeling and interpretation of the sub-techniques using video analysis or other data sources.

The literature on sub-technique classification methodology based on inertial measurement unit (IMU) sensors can mainly be divided into two groups. The first group is mechanism-driven, where the classification is based on general understanding of the dynamics, kinematics and other descriptions of the sub-techniques. See for example the following studies where this approach is employed: (Myklebust et al., 2011, 2015; Sakurai et al., 2014, 2016; Marsland et al., 2015, 2017, 2018; Seeberg et al., 2017). The second group consists of models based on expert-driven learning, where classification is based on supervised machine learning models trained by data sets labeled by experts (Holst and Jonasson, 2013; Stöggl et al., 2014; Rindal et al., 2017; Jang et al., 2018). These approaches can also be combined by considering labeled data and machine learning for calibration of the parameters in the mechanism-based methods or by using selected features instead of raw data in the training process of the supervised machine learning models. Independent of the approach, common definitions of the sub-techniques should be established and used in order to gain a proper comparison of the results from different approaches. Examples where ambiguity occur with today's sub-technique definitions are in: The transition phase between, or cycles containing, two different sub-techniques; new motion patterns that are significantly different from the established sub-techniques; and direction-changing motion patterns that occur in turns, track changes or during evasive actions. In the mechanism-based approaches, the common definitions need to be incorporated directly in the model definition, while the machine learning methods need data labeled according to the same detailed definitions. In the post-analysis of the results proposed by any of the classification methods, detailed insight into the sub-technique mechanisms may be gained. Incorporating these insights as they are discovered in different implementations (sensors and methodology) may gain a more unified and precise definition basis for discussing and classifying the sub-techniques.

Previously, in Seeberg et al. (2017), we demonstrated the possibility of using a multi-sensor system with time-synchronized multiple tri-axial IMU sensors placed on the arms (wrist) and lower legs (ankle) combined with heart rate (HR) and global positioning data to detect sub-technique distribution during classical XC skiing on snow. In a follow-up study (Rindal et al., 2017), we used the same sensor system framework with data from IMU sensors placed on the sternum and one arm (wrist) to develop an automatic classification of the sub-techniques based on machine-learning techniques. Finally, we have utilized a similar sensor system setup, with IMU sensors placed on the skis (in front of binding) and arms (wrist), to investigate sex-based differences in speed, sub-technique selection, and kinematic patterns during training for classical XC skiing across varying terrain (Solli et al., 2018). In the current paper we aim to bridge some of the gaps in these previous studies and to improve the comparability between the different classification methods in the literature in general.

This paper proposes a framework where basic motion components are used to specify the XC classical style sub-techniques in more detail. This framework formalizes the essential motion components, constructs non-intersecting sets of sub-techniques based on these components, and also proposes a selection of estimators for each of the components. Under the assumption that the proposed detailed specification of sub-techniques are accepted as definitions and that each of the components are perfectly measured, a theoretical proof declaring unique sub-technique classes is in principle sufficient validation of the approach. However, since the motion components are only estimated through models and real sensor data, our approach is also validated by a pilot data set utilizing labels independent of the proposed definitions. Furthermore, a classification method using sensors on arms and legs/skis was employed to describe the TRN and HRB sub-techniques in more detail than in previous approaches.

## 2. Methodology

The foundation of the proposed approach is to define a set of measurable motion components that can fully describe the distinguishable sub-techniques in a given sport. This concept is general and may be applied in any sport with cyclic motion patterns. The approach consists of:

- Motion and technique definitions, describing the basic observable motion components and the sport's techniques/sub-techniques
- Technique classification: The composition of the motion components that lead to unique technique/sub-technique decision functions and associated tolerance parameters
- Motion estimation: The implementation and realization of the motion components

The two first bullet points require fundamental knowledge of motion patterns typical for the considered sport and should be verifiable by researchers within the field. The collection of defined motion components may vary with respect to sensor types and placement. It will also vary with respect to the expert's qualitative evaluation of the sub-techniques, focusing on different aspects of the motion patterns in order to most efficiently separate the sub-techniques, i.e., identifying and formulating the motion components that provide the best balance between sensitivity and robustness with respect to each individual sub-technique. The core of the approach is to build on these motion components and show that resulting technique/sub-technique decision functions and tolerance parameters exist that produce unique technique/sub-technique classifications.

The terms sensitivity and robustness also reflect on the tolerance parameters, which need to be set such that the effects from the imperfections of the sensor systems and estimator are minimized. The sensor systems themselves introduce measurement errors in terms of bias and scaling factors, drift, noise, and precision limitations. In addition, the estimator may bring in modeling errors as the motion components are not necessarily measured directly. Thus, the selection and setting of the tolerance parameters are important for the performance and handling of uncertainty. As the parameters are integrated in the technique definitions, the tuning can be handled by the definition process and specified by a domain expert. Another approach to handle this is to formulate an optimization/calibration program, solved with machine learning or adaptive methods, relying on labeling of training/validation data sets. In the reminder of this section the methodology is applied to the case of classifying classical XC skiing sub-techniques.

### 2.1. Motion and Sub-technique Definitions

**Figure 1:**
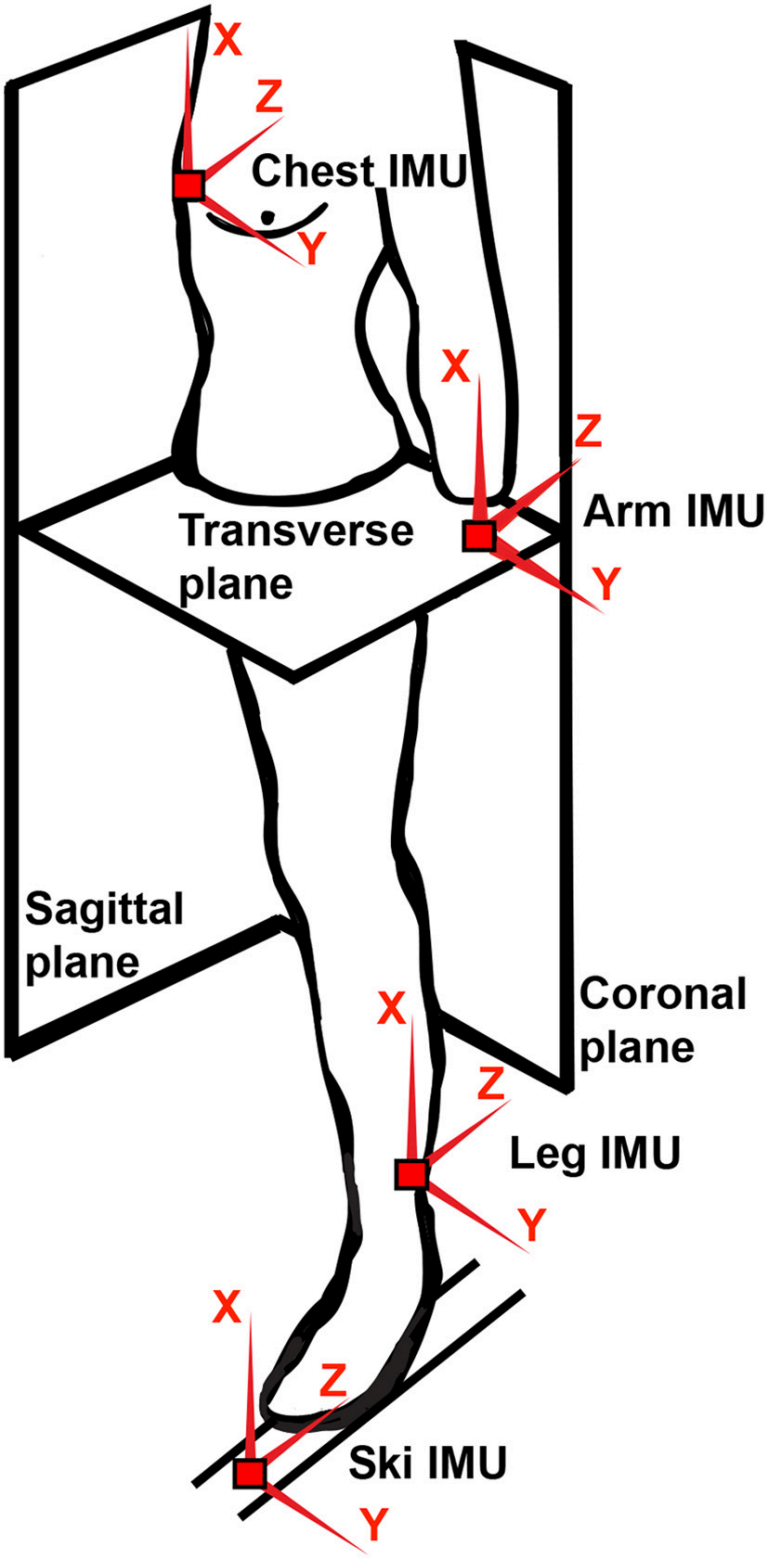
Illustration of body coordinates and placement of the IMU sensors. Note that the IMU sensors are depicted for the purpose of relating the case-study in section 3 to the diagram. However, other information sources such as camera-based systems may also be considered.

Definition 1. *Motion components and measures for classical XC skiing. For a time window Δ > *techCycle*, where techCycle is the time the athlete uses to perform a technique cycle, the following motion component definitions can be evaluated*:

1. *Arm synchronization: Level of synchronous arm motion around a common axis *a*_*lateral*_ defined in body coordinate frame, *y* in Figure 1, measured by for example correlation: armCorr [–1, 1]*.
2. *Independent leg motion: Level of independent leg sagittal motion measured by the function *legMoS*(*leg*_*workS*_)* :[0, *a*] → [0, ∞] *of class *K*. That is, *legMoS* is continuous, strictly increasing and *legMoS*(0) = 0. Here *leg*_*workS*_ represents the independent energy or displacement of the leg motions in the body sagittal plane. There is also a function *legMoST*(*leg*_*workST*_) that exhibits the same properties as the independent leg sagittal motion measure, but here the *leg*_*workST*_ represents the energy or displacement of the independent leg motion in the sagittal and transversal planes*.
3. *Arm motion: Level of arm motion measured by the function *armMo*(*arm*_*work*_) that exhibits the same properties as the leg motion measures*.
4. *Kick direction: The foot kick motion direction around the axis *a*_*vertical*_ defined in body coordinate frame, *x* in Figure 1. Measured by the cycle average rotation around *a*_*vertical*_: kickDir*.
5. *Kick rotation: The foot kick rotation around the axis *a*_*vertical*_ defined in body coordinate frame, *x* in Figure 1. Measured by a function of *K*_∞_, *kickRot*(*leg*_*rotwork*_) where *leg*_*rotwork*_ represents the energy or relative angular displacement of the leg motion rotation around *a*_*vertical*_*.
6. *Foot/ski orientation: The foot/ski orientation* Θ *relative to the body coordinate frame measured by the angles ϕ_*xx*_, θ_*xx*_ and ψ_*xx*_ where *xx* ∈ [*foot, ski*] around the axes *z, y, x* in Figure 1.*

*The motion component measures are lumped in a vector *x*_*motionComp*_ defined by the following*:

(1) $$\begin{array}{l} x_{motionComp}:=\left[legMoS,legMoST,armMo,armCorr,kickDir,kickRot,\psi_{ski}\right]\in \\ \;{\mathbb{R}}_{>}\times {\mathbb{R}}_{>}\times {\mathbb{R}}_{>}\times \left[-1,1\right]\times \left[-\pi ,\pi \right]\times {\mathbb{R}}_{>}\times \left[-\pi ,\pi \right] \end{array}$$

Definition 2. *Classical XC skiing sub-techniques*

1. *DIA: Arms and legs are active, and opposite leg and arms are in-phase synchronous while the arms are anti-phase synchronous. Ski orientation is kept in the longitudinal direction. Arm or leg motion defines the start and stop of the cycles*.
2. *DP: Poling with in-phase synchronous arm motion and insignificant independent leg motion. Arm motion defines the start and stop of the cycles*.
3. *Rotational kick (rK): A significant kick with a significant rotation around the vertical axis. The active leg motion defines the start and stop of the cycles*.
4. *DK: Poling with in-phase synchronous arm motion with a significant kick that is not defined as a rK. Arm motion defines the start and stop of the cycles*.
5. *HRB: Same as DIA but skis are rotated outwards in the opposite direction around the vertical axis. Arm or leg motion defines the start and stop of the cycles*.
6. *Double poling with a rotational kick (DPrK): Poling with in-phase synchronous arm motion and a rK. Arm motion defines the start and the stop of the cycles*.
7. *noTech: Any skiing activity not defined by the bullets above*.

Definition 2 represents the authors' qualitative description of the classical XC sub-techniques. It relates to the FIS International Competition Rules (ICR) 310.2.2^1^ from the FIS website^2^, but describes the movement patterns more explicitly.

Remark 1. *Excluded activities*

- *Activities where an athlete is performing skating style sub-techniques, skiing without poles, running with poles, bare running or performing any other activity that could produce motion patterns similar to the definitions are not considered valid in this context*.
- *Additional motion components may be included to provide a more specific description of the sub-techniques. Specific examples are components related to ski gliding and the diagonal synchronicity between arms and legs to also classify running and amble gait*.
- *Non-cyclic activities like downhill tucking, standing still and non-repetitive motion are not standard classical XC skiing sub-techniques and will be covered by the noTech class. However, these activities are included here in order to fully span the classification sample space*.

### 2.2. Sub-technique Identification

Assumption 1. *The athlete performs classical XC skiing, and the variables *x*_*motionComp*_, representing the chosen measures from Definition 1, are independent*.

Proposition 1. *Under Assumption 1, the motion components from Definition 1 are sufficient measures for a unique decision function description of the classical XC sub-techniques in Definition 2*.

*Proof*: In the following, *x* = *x*_*motionComp*_ is used to allow a more compact notation. The outline proof is given by proposing the following logical compositions, derived from the definition 2, as decision functions:

(2) $$\begin{array}{l} l_{DParm}\left(x\right):=\left(armMo>tol_{armMo}\right)\wedge \left(armCorr>tol_{armPole}\right),\\ \;\in \left[0,1\right] \end{array}$$

(3) $$\begin{array}{l} l_{HRBDIA}\left(x\right):=\left(armMo>tol_{armMo}\right)\wedge \left(armCorr<tol_{armDiag}\right),\\ \;\in \left[0,1\right] \end{array}$$

(4) $$\begin{array}{l} l_{HRB}\left(x\right):=l_{HRBDIA}\left(x\right)\wedge \left(kickDir>tol_{kickDir}\vee \psi_{ski}>tol_{\psi }\right),\\ \;\in \left[0,1\right] \end{array}$$

(5) $$\begin{array}{l} l_{DIA}\left(x\right):=l_{HRBDIA}\left(x\right)\wedge \neg l_{HRB}\left(x\right)\wedge \left(legMoS>tol_{legMoS}\right),\\ \;\in \left[0,1\right] \end{array}$$

(6) $$\begin{array}{l} l_{DPrK}\left(x\right):=l_{DParm}\left(x\right)\wedge \left(kickRot>tol_{kickRot}\right)\\ \;\wedge \left(legMoST>tol_{legMoST}\right),\in \left[0,1\right] \end{array}$$

(7) $$\begin{array}{l} l_{DK}\left(x\right):=l_{DParm}\left(x\right)\wedge \left(legMoS>tol_{legMoS}\right)\wedge \neg l_{DPrK}\left(x\right)\\ \;\wedge \left(legMoS>tol_{legMoS}\right),\in \left[0,1\right] \end{array}$$

(8) $$\begin{array}{l} l_{DP}\left(x\right):=l_{DParm}\left(x\right)\wedge \left(legMoST<tol_{legMoST}\right)\\ \;\wedge \left(legMoS<tol_{legMoS}\right),\in \left[0,1\right] \end{array}$$

(9) $$\begin{array}{l} l_{rK}\left(x\right):=\left(kickRot>tol_{kickRot}\right)\wedge \left(armMo<tol_{armMo}\right)\\ \;\wedge \left(legMoST>tol_{legMoST}\right),\in \left[0,1\right] \end{array}$$

**Figure 2:**
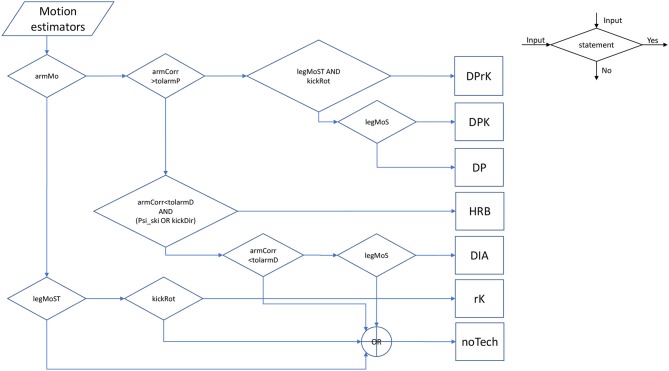
An example flowchart of the decision functions from Equation (3) to (9). With the exception for *armCorr* the tolerances are omitted in the diagram in order to reduce the notation.

Let all tolerance variables: *tol*_*armDiag*_, *tol*_*armPole*_, *tol*_*legMoS*_, *tol*_*legMoST*_, *tol*_*armMo*_, *tol*_*kickRot*_, *tol*_ψ_, *tol*_*kickDir*_, be within the domain of the comparable motion measures. Then since *x* are independent measures, the sets {*x* ∣ *l*_*i*_(*x*) = 1}, ∀*i* ∈ *A*: = {*HRB, DIA, DP, DPK, DPrK, rK*} are pair-wise disjoint by construction, see Figure 2 for an overview of the decision flow—i.e., {*x* ∣ *l*_*j*_(*x*) = 1} ∩ {*x* ∣ *l*_*i*_(*x*) = 1} = ∅∀*i* ≠ *j* ∈ *A*. The non-technique set is defined by:

(10) $$\begin{array}{l} l_{noTech}:=\neg \left(\vee_{j\in A}l_{j}\left(x\right)\right),\in \left[0,1\right] \end{array}$$

Thus, the sets $\left\{x|l_{i}\left(x\right)=1\right\},\forall i\in A^{*}:=A\cup \left\{noTech\right\}$ are also pair-wise disjoint, and Equations (3)–(10) are unique decision function descriptions that represent the classical XC skiing sub-techniques in Definition 2.

Remark 2. *Note that Proposition 1 only has a “sufficient” claim, and that this claim is two-fold. First, this means that there may exist different motion component measures that can be used in the sub-technique decision functions. Second, since Definition 2 only holds qualitative information, other decision function definitions may be proposed, i.e., the realization of Definition 2, represented by the decision functions from the proof may be changed with other measures and function definitions*.

Remark 3. *The structure of the proposed decision function relies on three “super” classes derived from the arm motion component: Correlated, anti correlated and no-arm motion. All sub-techniques belong to either of these classes and are further refined by the leg motion components*.

Remark 4. *The decision functions *l*_*j*_ and the specific set of tolerance parameters in proof of Proposition 1 may be used as a detailed measurable definition of the classical XC sub-techniques. A drawback of such an approach is that this would require models and sensor systems that specifically estimate the proposed motion components. This can however be handled by standards, specifying motion components and decision functions dependent on the sensor setup*.

Remark 5. *Note that the requirement in Definition 1 Δ*t* > *techCycle*, may be conservative when considering a fixed window for all sub-techniques, all sessions and all athletes. If a sub-technique cycle consists of two anti- or in-phase motion patterns, and the motion components do not discriminate between the two, then the time window can be relaxed to* $\Delta t>\frac{techCycle}{2}$ *for that particular sub-technique. This is the case for DIA and HRB. In Definition 2, DK is defined independent of which leg generates the kick, such that the DK cycle is constrained by the arm cycle time*.

### 2.3. Motion Estimators

The quantification of the motion components in Definition 1 relies on measurements from sensor systems either in the environment or being attached to the athlete or the equipment. In this work, IMU sensors were attached to the arms and skis of the athlete to provide acceleration and angular rate information that were used to estimate the motion components. The term “estimators” is used here since the components were indirectly estimated through a model relying on certain assumptions, or since signal processing on the sensor raw data (like filtering) were necessary due to sensor measurement errors and noise.

Assumption 2. *Sensor calibration. All sensor information is time synchronous and aligned to a common athlete body coordinate frame, defined in Figure 1*.

#### 2.3.1. Arm Synchronization

To obtain a measure of the arm synchronization level, the correlation coefficient for each time-stamp *t* over the window Δ*t* related to *techCycle* is used here and calculated as follows:

(11) $$\begin{array}{l} ss_{xy}\left(t,\Delta_{t}\right):=\underset{i\in \left[t-\frac{\Delta t}{2},t+\frac{\Delta t}{2}\right]}{\sum }\left(x_{i}-\bar{x_{t}}\right)\left(y_{i}-\bar{y_{t}}\right) \end{array}$$

(12) $$\begin{array}{l} armCorr\left(t,\Delta_{t}\right):=\frac{ss_{A_{left}A_{right}}\left(t,\Delta_{t}\right)}{ss_{A_{left}A_{left}}\left(t,\Delta_{t}\right)ss_{A_{right}A_{right}}\left(t,\Delta_{t}\right)} \end{array}$$

To simplify calibration and maximize the signal to noise ratio, gyroscope sensors were used as comparison signals *A*. Here *A*_*left*_ and *A*_*right*_ are the angular rates around the **lateral axis** from the left and right arms respectively. Note that more elaborated approaches utilizing all the channels of the accelerometers and gyroscopes may be considered.

#### 2.3.2. Leg and Arm Motion

As for arm synchronization, the angular rate around the arm's lateral axis was used as a basis for estimating arm motion. From the sum of squared values in Equation (11), the variance of the arm motion components can be calculated:

(13) $$\begin{array}{l} \sigma_{x}^{2}\left(t,\Delta_{t}\right)=\;\frac{ss_{xx}\left(t,\Delta_{t}\right)}{fr\Delta_{t}} \end{array}$$

(14) $$\begin{array}{l} armMo\left(t,\Delta_{t}\right):=\;\sigma_{A_{right}}^{2}\left(t,\Delta_{t}\right)+\sigma_{A_{left}}^{2}\left(t,\Delta_{t}\right) \end{array}$$

where σ(*t*, Δ_*t*_) denotes the standard deviation at time *t* over a window of Δ_*t*_ and *fr* is the sampling rate of the signal.

For the independent leg motion estimation, the difference in relative angles of the legs is used as a basis. These signals are derived from the angular rate around the lateral and vertical body axes. The angular rate raw data are band-pass filtered, integrated with bias removal and finally differentiated accordingly:

(15) $$\begin{array}{l} r_{FLH}\left(t\right)=\;bandpass\left(lowB,highB,r_{F}\left(t\right)\right) \end{array}$$

(16) $$\begin{array}{l} \Theta \left(t\right)=\;\int r_{FLH}\left(t\right)dt \end{array}$$

(17) $$\begin{array}{l} \Theta_{M}\left(t\right)=\;\Theta \left(t\right)-mean\left(\Theta \left(t\right)\right) \end{array}$$

(18) $$\begin{array}{l} angDiff_{\Theta_{R}}\left(t\right):=\;\Theta_{Mleft}\left(t\right)-\Theta_{Mright}\left(t\right) \end{array}$$

where *r*_*F*_ and *r*_*FBH*_ represent the measured and filtered rotational rates, and Θ and Θ_*M*_ are the estimated and bias removed orientation of the legs. Here *angDiff*_Θ_(*t*) represents the angular difference between the left and the right leg. The independent leg motion is then estimated similar to Equation (13) given by:

(19) $$\begin{array}{l} legMoS\left(t,\Delta_{t}\right):=\;\sigma_{angDiff_{\theta_{R}}}^{2}\left(t,\Delta_{t}\right) \end{array}$$

(20) $$\begin{array}{l} legMoST\left(t,\Delta_{t}\right):=\;\sigma_{angDiff_{\theta_{R}}}^{2}\left(t,\Delta_{t}\right)+\sigma_{angDiff_{\psi_{R}}}^{2}\left(t,\Delta_{t}\right) \end{array}$$

Where θ_*R*_ and ψ_*R*_ denote the angle differences around the lateral and vertical axes respectively.

#### 2.3.3. Kick Rotation

Several methods may be applied to quantify the athlete's kick rotation. For example, any norm of the angle around the vertical axis over the time period Δ*t* could be used. In this presentation, the signal strength of the rotation around the vertical axis is compared with the rotation around the lateral axis. This was chosen such that the tolerance parameter was less influenced by the individual differences in athlete capabilities.

(21) $$\begin{array}{l} kickRot\left(t,\Delta t\right):=\frac{\sigma_{angDiff_{\psi H}}\left(t,\Delta_{t}\right)}{\sigma_{angDiff_{\theta H}}\left(t,\Delta_{t}\right)} \end{array}$$

Note that $kickRot(t,\Delta t)\;\overset{\sigma_{angDiff_{\theta H}}(t,\Delta_{t})\;\to \;0}{\to }\;\infty$. This is handled by the leg motion restrictions, preventing rotational kick classification in cases of insignificant motion.

#### 2.3.4. Foot/Ski Orientation

Typically angular rate measurements provide a good signal to noise estimation of orientation, but at the cost of estimator drift due to bias and numerical issues during the integration step, i.e., the absolute orientation is not available. Due to the gravitational field, the accelerometer data may be used to generate absolute estimates of angles around the longitudinal and lateral axis, under the assumption of relative low dynamic environment. Combining the two measurement sources is common in navigational and robotic applications, see for example Fossen (2002) or Grøtli et al. (2016), producing high resolution and precision attitude estimates. However, the main requirement in the following section HRB estimator is the availability of absolute estimates, i.e., the angular rate measures are in this context less relevant. Absolute yaw/heading estimates may be provided by magnetometers or a dual antenna global navigation satellite system (GNSS) setup, using the ski as a baseline. However, neither of these sensor systems were available in this work, thus only absolute roll and pitch estimates are provided. These can be estimated through knowledge of the gravity components, see for example the textbook by Farrell and Barth (1999), and calculated according to the following relationship:

(22) $$\begin{array}{l} f=\left[\begin{array}{c} \sin\theta \\ -\cos\theta \sin\phi \\ -\cos\theta \cos\phi \end{array}\right]g \end{array}$$

(23) $$\begin{array}{l} f_{f}\left(t,\Delta t_{skiOri}\right):=\;\frac{1}{\Delta t_{skiOri}}\underset{i\in \left[t-\frac{\Delta t_{skiOri}}{2},t+\frac{\Delta t_{skiOri}}{2}\right]}{\sum }f\left(i\right) \end{array}$$

(24) $$\begin{array}{l} \phi \left(t,\Delta t_{skiOri}\right)=\;atan2\left(-f_{fy},-f_{fz}\right) \end{array}$$

(25) $$\begin{array}{l} \theta (t,\Delta t_{skiOri})=\;atan2(f_{fx},\sqrt{f_{fz}^{2}+f_{fy}^{2}}) \end{array}$$

where *g* is the gravity component and *f* represents the acceleration measurements. The use of the absolute angular estimates of the skis in this work is limited to the low pass components (*f*_*f*_). Therefore, a moving average filter with a window Δ*t*_*skiOri*_ was used to remove the high frequency components of *f*(*t*).

#### 2.3.5. ψ_*ski*_ E Estimator and Kick Direction

HRB can be identified by using the ski orientation or the kick direction as estimators. The kick direction may be identified by acceleration measurements from the skies but this has not yet been explored and is left for further work. The preferred estimator of HRB is the difference in ski yaw/heading. For example in Andersson et al. (2014b) the HRB technique was video analyzed and characterized at an incline of 15 degrees. The athletes employed in this study a lateral angle/yaw between the skis at mean values of 25–30 degrees and standard deviation between 4 and 11 degrees, both values increasing with lower velocity. In our presentation reliable estimates of the absolute yaw were not available as there was no magnetometer in the applied IMU sensors. Absolute ski roll and pitch are however estimated by using the gravitational acceleration component as a reference. Note that these estimates differ from the relative angular estimates presented in Equation (15), which were based on the angular rate measurements from the IMU. It is common to combine the two estimates in an attitude observer, as discussed in section 2.3.4. For simplicity this is not considered as it will not gain any principal advantages for the presented low frequency estimator. However, in cases with absolute yaw measurements available and estimators utilizing high frequency components, such observers should be considered. The HRB estimator used in this study was defined by:

(26) $$\begin{array}{l} e\psi_{ski}:=\;\left(\phi_{left}-\phi_{right}\right)\left(\theta_{left}+\theta_{right}\right) \end{array}$$

(27) $$\begin{array}{l} l_{HRBDIA}\left(x\right)=\;\left(armMo>tol_{armMo}\right)\wedge \left(legMo>tol_{legMo}\right)\\ \;\wedge \left(armCorr<tol_{armDiagHrb}\right) \end{array}$$

(28) $$\begin{array}{l} l_{HRB}\left(x,\phi ,\theta \right)=\;l_{HRBDIA}\left(x\right)\wedge \left(e\psi_{ski}>tol_{e\psi }\right) \end{array}$$

(29) $$\begin{array}{l} l_{DIA}\left(x\right)=\;l_{HRBDIA}\left(x\right)\wedge \neg l_{HRB}\left(x\right)\wedge \\ \;\left(armCorr<tol_{armDiagD}\right) \end{array}$$

where *eψ*_*ski*_ is the ski orientation estimate that replaces *kickDir* and ψ_*ski*_ in Equation (4). ϕ and θ are the ski pitch and roll angles in radians. Furthermore, notice that the negative arm correlation constraint is made more strict for the DIA sub-technique with *tol*_*armDiagD*_ < *tol*_*armDiagHrb*_, in order to have a more robust separation between the two sub-techniques (DIA and HRB). The main component in the *eψ*_*ski*_ estimate is the ski roll angle. Under the assumptions that the roll angles are relatively small and that the athlete keeps the skis in parallel and legs straight with stiff ankle and knee, then the roll angle will measure the distance between the skis:

(30) $$\begin{array}{l} legLatDist=hipWidth+\left(\phi_{left}-\phi_{right}\right)legHight \end{array}$$

This estimate may be used for setting a reasonable *tol*_*eψ*_ coefficient. Including the pitch (θ) angles in the estimator and a positive tolerance parameter ensures that HRB will only be classified in uphill terrain. Turning and skating downhill will produce negative estimates.

## 3. Case Study

**Figure 3:**
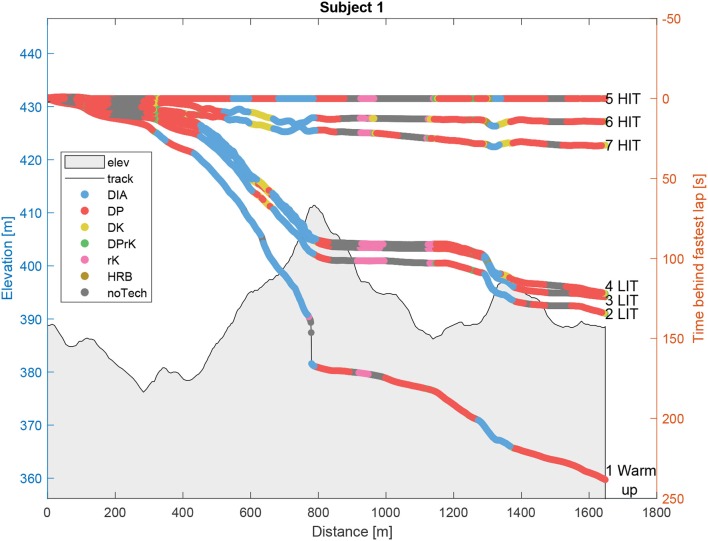
Time behind the fastest lap relative to the distance from the start of the lap. The fastest lap (5) is used as a reference and all data are projected onto this lap through linearly scaled distance measures. The specific sub-techniques and the elevation are displayed along the distance to show the elevation effects. Lap 1 (Warm-up), 2–4 (Low-intensity training, LIT), and 5–7 (High-intensity training, HIT).

**Figure 4:**
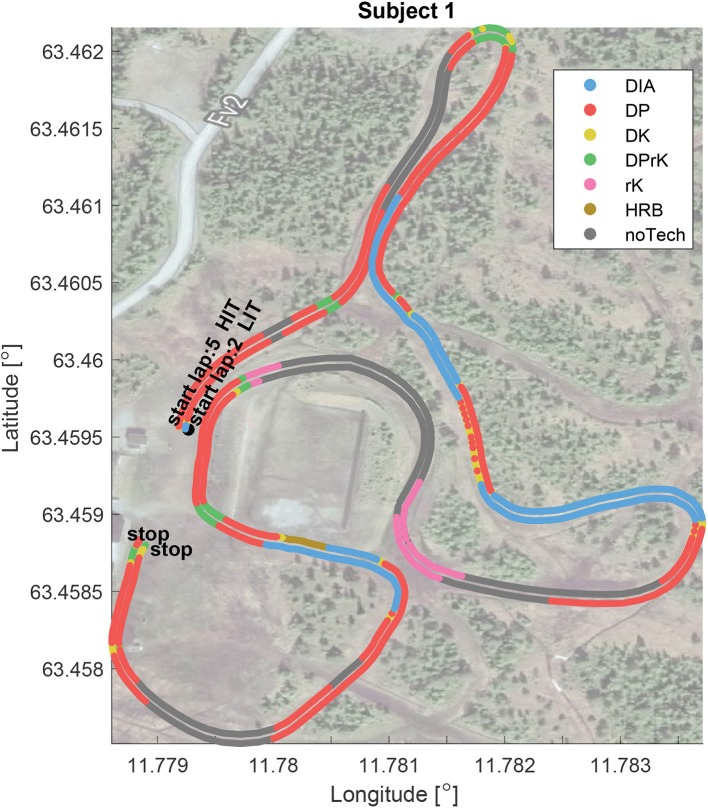
Positional sub-technique distribution. Comparing the sub-technique distribution of the low-intensity (LIT) validated lap 2 with the fastest high-intensity (HIT) lap 5. The HIT lap is projected onto the LIT lap and shifted 0.00006° outwards in order to visualize the relative differences. The plot highlights the sub-technique distribution with dependence on the track turns.

In order to demonstrate the framework, data from an outdoor field trial at Meråker in 2017 was used (Solli et al., 2018). All results and plots were generated by MATLAB analysis tools, among them *skiViewer*, developed in the two projects AutoActive and emPower, both supported by the Norwegian Research Council. The study was pre-approved by the Norwegian Centre for Research Data, conducted in accordance with the Declaration of Helsinki and assured by the responsible institution, the Norwegian University of Science and Technology. All participants were fully informed of all test protocols and procedures before they provided their written consent to participate. The main objective of the Meråker study was to combine HR monitoring, GNSS, and micro-sensor technology to investigate sex-based differences in speed, sub-technique selection, and kinematic patterns during low-intensity training (LIT) and high-intensity training (HIT), where LIT: HR < 82% of maximal HR (HR_*max*_) and HIT: HR > 87% of HR_*max*_, for classical XC skiing across varying terrain. The skiers were instructed to initially ski at a low intensity using their preferred sub-technique, then at competition speed (i.e., HIT), with approximately 2 min of rest in between. The same course was used for both the LIT and HIT tests, consisting of three rounds of a 1.7 km-long track with varying elevation and turn topology. The track represents a typical racing track, and it was chosen to stimulate the athlete to use their full repertoire of sub-techniques. In Figures 3, 4, an overview of the course elevation and map positions is shown and decomposed by comparable segments, starting, and stopping at the same positions for each round. To compare the algorithm results with reference data, two men and two women were randomly selected. These skiers are referred to as subjects 1 to 4 throughout the text. All test subjects had competed at national and international levels, and the use of sub-technique in the LIT session was labeled and manually synced to the motion data based on a video, captured by a skier following the test subject. The labeling was done independent of the definitions in this paper. The sub-technique cycles were defined to start and stop when the subject's left arm was extended all the way behind the body (Rindal et al., 2017). Each cycle was then labeled either DIA, DK, DP, HRB, TRN, TCK, transition to DIA (tDIA), and transition from DIA (fDIA)

To establish a proper comparison between the expert qualitative evaluation (labeling of the sub-techniques) and the definitions in paper, a set of rules/mappings of the labels to a common reference set were made.

Definition 3. *Comparison rules*

- Converting from data samples to cycles: Within each cycle, the sub-technique with the highest sum of algorithm classification samples was chosen to represent the algorithm classification result. Note that this will have some low pass filtering properties, letting the majority of samples represent the cycle
- *Turn sub-techniques: The two sub-techniques DPrK and rK were merged and labeled to the TRN class*.
- *Tucking: The TCK class appears between cycles and is not considered a sub-technique cycle in this paper. It is therefore labeled as noTech*.
- *Transition techniques: Two mappings were considered*.
  – a) The first maps tDIA and fDIA to DIA
  – b) While the second maps tDIA as DIA but fDIA as DK
    *See section 4.3 for a detailed discussion on the implications*.

These mapping rules were based on discussions between XC skiing experts and algorithm developers. Discussions like these are practical examples of why it is necessary to work toward a common framework for motion components and the unique sub-technique definitions. Further discussion of the rules is provided in section 4.3.

### 3.1. Instrumentation

This work builds upon data presented in Solli et al. (2018), where motion data were collected by six IMU sensors (Physiolog 5, GaitUp, Switzerland), consisting of a triaxial accelerometer and gyroscope and a barometric pressure sensor. The sensor system sampling frequency, 256 Hz, was down sampled to 20 Hz and all data channels were synchronized in time, before the classification. The IMU sensors were mounted using straps with velcro on the body—the sternum, lower back, and wrists—and with velcro straps in front of the binding on the left and right skis (Figure 1). The reason for placing the sensors on the skis was to collect data of the ski-motion directly. Garmin Forerunner 920XT (Garmin Ltd., Olathe, KS, USA), with multi constellation GNSS active (GPS and GLONASS) and barometric altitude monitor, validated in Gløersen et al. (2018), was included in the system and used to measure the position, HR and altitude with a sampling frequency of 1 Hz. The positioning system was mainly used for comparing the identified techniques throughout the track. Hence, a frequency of 1 Hz was sufficient for segment definitions under the assumptions that the segments were sufficiently long. Video was captured during the LIT laps using a Garmin VIRB (Garmin Ltd., Olathe, KS, USA) placed on the forehead of a skier following the test subjects. All data were logged on the individual sensor systems during the tests and analyzed offline in MATLAB (MathWorks, Natick, MA, USA). The GNSS and IMU data were synchronized by high-pass filtering and cross-correlating data were recorded by the barometer in both sensor systems. In order to normalize differences in sensor positioning, the arm sensors were calibrated by following Seeberg et al. (2017), and the ski-mounted sensors were aligned by assuming zero average horizontal acceleration throughout the activity.

### 3.2. Algorithm Parameters

The sampling and tolerance parameters used in the classification algorithm implementation are given in Table 1, related to sections 2.3.1 and 2.3.4, and Table 2, related to proof of Proposition 1 and section 2.3.5. The parameters were mainly set by assuming a typical sub-technique cycle length and manual inspection of the data sets. The parameter Δ*t* may be considered time varying and is identified by frequency analyzing the arm motion throughout the session, as presented by Rindal et al. (2017). An analysis of the performance sensitivity of this parameter was not conducted in this study, but large values are expected to produce classification errors in transition phases, while small values may produce errors due to lack of discriminating information.

**Table 1:** Algorithm and estimator sampling parameters.

**Data windows and sampling parameters**				
**Analysis window Δ*t* [*s*]**	**Absolute angle region Δ*t*_*skiOri*_ [*s*]**	**Sampling frequency [*s*^−1^]**	**Pass low band lowB [*s*^−1^]**	**Pass high band highB [*s*^−1^]**
1.3	2.5	20	0.3	3

**Table 2:** Algorithm and estimator tolerance parameters.

**Tolerance parameter values from the proof of proposition and estimators**				
*tol*_*armDiagD*_	*tol*_*armDiag*_	*tol*_*armPole*_	*tol*_*legMoST*_	*tol*_*legMoS*_
–0.4	–0.3	0.4	9^2^	1.5^2^
*tol*_*armMo*_	*tol*_*kickRot*_	*tol*_*e*_ψ__*ski*__	*tol*_*armDiagHrb*_	
1e4	2	0.06	–0.3	

## 4. Results and Discussion

In this section, the results from the implementation of the sub-technique classification approach, section 2, is presented and discussed based on data produced by the case study presented in section 3. The discussions underline the motivation for developing a more detailed platform for comparing technique analysis methods, detailing the motion components, preventing unambiguous definitions and allowing more precise discussions and quality assessments of an athlete's technical ability. Such understanding is highly relevant for providing a valid communication platform for researchers in this field, as well as for developing preparation and debriefing tools for coaches and athletes as exemplified below.

### 4.1. Technique Distribution Overview

Table 3 and Figures 3, 4 show the sub-technique distribution for a representative participant's (i.e., Subject 1) session, calculated by the presented approach. The session had seven laps, one warm-up lap, three LIT video validated technique laps (2–4) and three HIT laps (5–7). The fastest lap was the first of the HIT laps marked “5” plotted at the reference zero “*Time behind fastest lap”* along the x-axis *Distance* in Figure 3. All other laps, and associated data, are projected onto the distance defined by the fastest lap, in order to allow a proper comparison. Figure 4 only contains the first LIT lap and the fastest HIT lap in the horizontal plane. This is done to simplify the view of the sub-technique distribution, dependent on intensity, position, and track curvature.

**Table 3:** Technique distribution together with lap and physiological parameters.

**Subject 1 performance summary**							
	**Warm-up**	**LIT**			**HIT**		
	**Lap: 1**	**Lap: 2**	**Lap: 3**	**Lap: 4**	**Lap: 5**	**Lap: 6**	**Lap: 7**
Time [*h* :*min* :*s*]	00:08:20	00:06:36	00:06:26	00:06:23	00:04:21	00:04:36	00:04:51
Total distance [*m*]	1680	1664	1669	1681	1649	1681	1670
Total climb [*m*]	60	59	61	60	54	55	55
Speed [*m* · *s*^−1^]	3.36	4.20	4.32	4.38	6.29	6.09	5.73
HR [*beats* · *min*^−1^]	123	138	135	135	168	178	180
HR/HR_*max*_[%]	63.7	71.5	69.9	69.9	87.1	92.3	93.3
Arm frequency(freq) [*s*^−1^]	0.71	0.67	0.66	0.67	0.85	0.82	0.84
DIA [%] (freq [*s*^−1^])	41 (0.77)	39 (0.77)	39 (0.77)	37 (0.78)	19 (0.96)	21 (0.93)	22 (0.95)
DP [%] (freq [*s*^−1^])	39 (0.74)	29 (0.70)	30 (0.69)	29 (0.72)	53 (0.96)	43 (0.95)	44 (0.93)
DK [%] (freq [*s*^−1^])	01 (0.64)	08 (0.65)	07 (0.63)	09 (0.65)	03 (0.96)	11 (0.75)	11 (0.80)
DPrK [%] (freq [*s*^−1^])	04 (0.72)	04 (0.75)	03 (0.75)	03 (0.80)	05 (0.94)	05 (1.00)	06 (0.99)
rK [%] (freq [*s*^−1^])	02 (0.62)	02 (0.55)	02 (0.32)	02 (0.36)	02 (0.27)	01 (0.25)	02 (0.33)
HRB [%] (freq [*s*^−1^])	00 (NaN)	00 (NaN)	00 (NaN)	01 (0.96)	02 (1.23)	02 (1.12)	01 (1.21)
noTech [%] (freq [*s*^−1^])	14 (0.47)	18 (0.40)	19 (0.40)	18 (0.36)	17 (0.37)	17 (0.34)	15 (0.41)
BLa_*peak*_ [*mmol* · *L*^−1^]				1.3			13
RPE [Borg 6 − 20]				11			18

*LIT, low-intensity training: Blood lactate concentration (BLa_peak_) < 2.5 mmol·L^−1^, rating of perceived exertion (RPE) < 14Borg or heart rate (HR) < 81% of maximal heart rate (HR_max_) HIT, high-intensity training: BLa_peak_ >4.0 mmol·L^−1^, RPE >16Borg, HR >87% of HR_max_*.

*DIA, DP, DK, DPrK, rK, HRB, and noTech denotes the sub-techniques from section 2. The table values are given by the ratio of the performed sub-technique samples in a lap with respect to all the samples in that lap*.

By listing the sub-technique from the lowest to highest gears, Figures 3, 4 show how the athlete uses HRB in the steepest part of the track (around 1,300 m); DIA in the moderate to steep hills (400–800 m); DK in moderate incline and transition between DIA and DP (for example 600–700 m); DPrK in turns in moderate to negative incline (at 350 m and track changing parts at approximately 600 m and toward the lap end); DP for example after the top of a hill; rK in the downhill parts, including turns; and finally noTech mainly in the samples where the athlete is racing downhill in the TCK position.

Table 3 summarizes the lap-timing, sub-technique distribution and physiological parameters for Subject 1, which provides a basis to the overall motivation for identifying the sub-technique distribution. It is an example on how information from different sensor systems may be used as a tool by coaches and athletes to evaluate a training session in more details. For example, the transition from LIT to HIT obviously results in higher HR and speed. But comparing the HIT laps internally shows an increased lap time with increased HR. Furthermore, the sub-technique distribution reveals that the subject shifted the use of high gear DP in the first HIT lap (5), to the active leg techniques DIA and DK in the two last HIT laps (6 and 7). Grouping this information may give insight to the athlete's pacing strategies and fatigue, but may also be a result of changes in equipment or other external factors, e.g., snow conditions. In this multiple IMU sensor setup, cycle frequencies are readily available features to be analyzed with the sub-technique distribution. For Subject 1, DIA, DP, and DPrK were performed at similar arm frequencies for the same intensities, but increased from the LIT to the HIT laps. The DK was performed at a lower frequency which is common, and HRB at a higher frequency which is due to steep hills and no ski gliding. The low arm frequency estimates in rK and noTech are less relevant since the arm motion in these techniques was not defined. The frequencies depicted in the table were calculated by cross-correlating the angular rate around each of the arms, and averaging the results. Further work will include the leg motion in the frequency estimation. This ability to collect and combine more detailed information about the session performance and context enables the coaches and athletes to evaluate their racing tactics, training planning, training sessions, skiing equipment, and generally aid researchers in testing new hypotheses coupling physiological, mechanical, and other relevant contextual information both in lab and field conditions (Marsland et al., 2017, 2018; Solli et al., 2018).

The quality of the performed sub-techniques is not evaluated in the classification process *per se*, and both “good” and “bad” sub-technique performance are classified in accordance with the tolerance values. However, increased tolerances and a narrower technique cycle window parameter will enlarge the *noTech* set and reduce the remaining sets of sub-techniques. Such parameter adjustments may be used for “filtering” quality sessions, evaluating the athlete time/distance usage of more “precise" sub-technique classes. This may be a useful tool for high-level XC skiing athletes who typically perform the sub-techniques in interval and race sessions more distinctly. If the goal is to record what “looks like" a sub-technique in a broader sense according to definition 2, comparing a variety of sub-technique implementations spanning recreational to high-level athletes, less restrictive tolerance parameters should be used. The presented results are based on data from high-level XC skiing athletes, but a set of less restrictive parameters was used. These parameters are similar to the tolerances used in Seeberg et al. (2017) for classification of DIA, DP, and DK where the study also included recreational athletes.

In recent years, the classical XC skiing sub-techniques have been extensively debated and new regulations have been introduced, restricting equipment (e.g., pole length) and sub-technique usage along the track (e.g., diagonal zones, where only one pole is allowed in the ground at any time). As such, automated or decision support tools for enforcing these rules require common qualitative detailed definitions of the sub-techniques and also the sub-techniques' distribution around the race track, as exemplified by Figure 3, 4.

### 4.2. Algorithm Mechanisms

**Figure 5:**
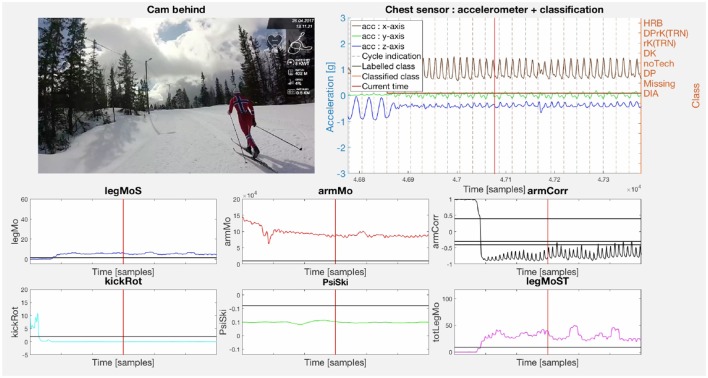
The figure displays a snapshot of the video of subject 1 synchronized with the sensor data and motion estimators. The video is in the first subplot. The second subplot shows the accelerometer data from the chest sensor plotted with the classification of each cycle according to Equations (5) to (10) and (28) together with the manual expert labeling. Subplots 3 to 8 show the motion estimators defined in Equations (19), (13), (11), (21), (24), and (20), respectively. The tolerance value of each motion estimator is plotted as the dark horizontal line in each subplot. The criteria for the DIA definition in Equation (5) is satisfied as is illustrated with (high *legMoS* and *armMo*, low *armCorr*, *kickRot*, and ψ_*ski*_) while simultaneously not satisfying the criteria for the HRB definition in Equation (28). The figure is a single video frame from a full video of the subjects synchronized with the sensor data and motion estimators. An example video is available as Supplementary Materials.

**Figure 6:**
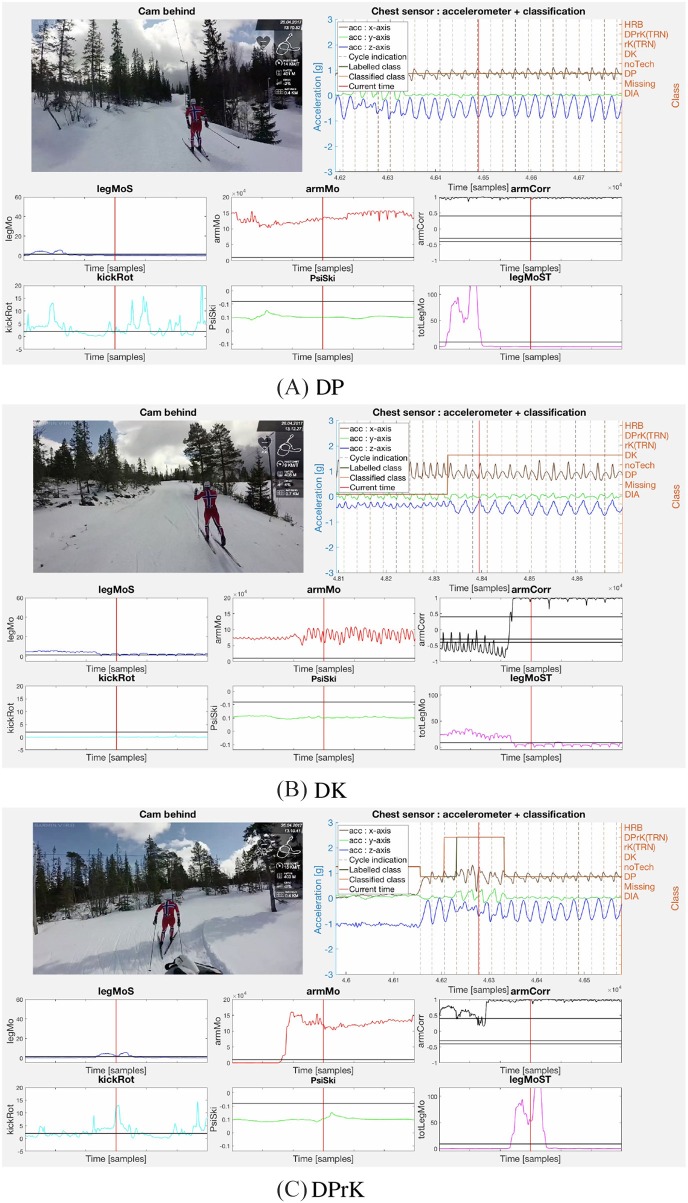
Caption **(A)** displays Subject 1 performing the DP sub-technique as defined by Equation (8) (high *armMo* and *armCorr*, low *legMoS*, and *legMoST*). The second caption, **(B)**, displays subject 1 performing the DK sub-technique as defined by Equation (7) (high *armMo*, *armCorr*, *legMoS*, and *legMoST*) but not satisfying the conditions for DPrK in Equation (6). DPrK is displayed in **(C)** by subject 1 (high *armMo*, *armCorr*, *kickRot*, and *legMoST*). An example video is available as Supplementary Materials.

**Figure 7:**
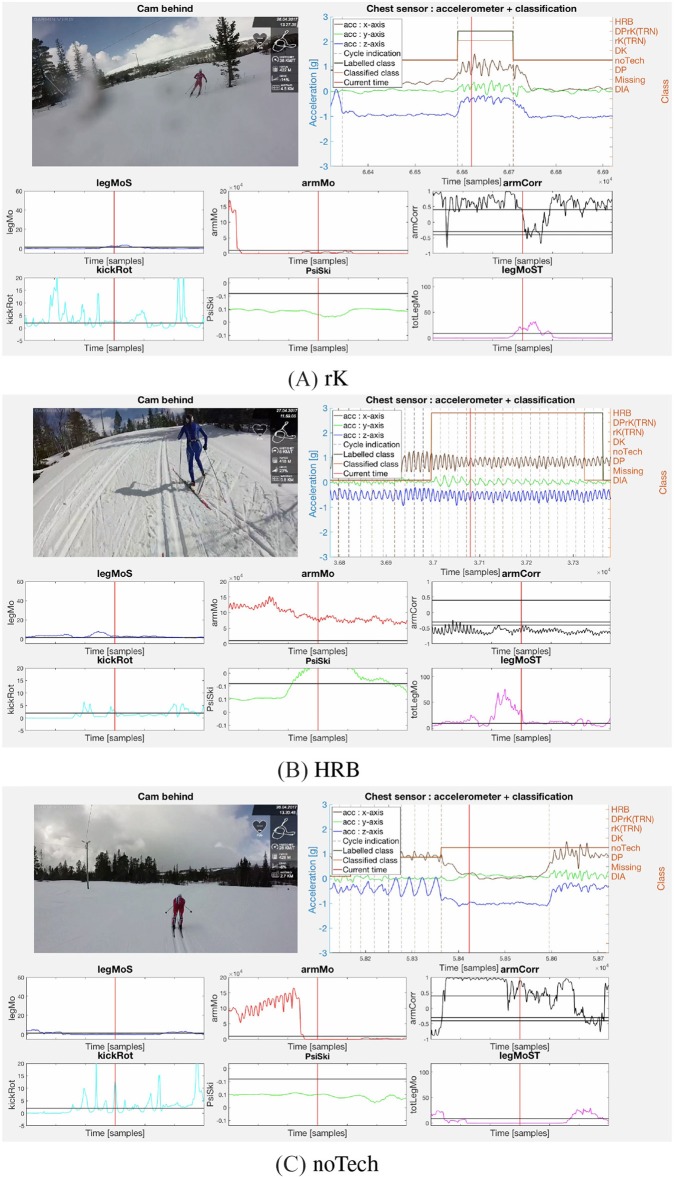
The rK sub-technique, performed by subject 1, is displayed in **(A)** with low *armMo* but high *legMoST* and *kickRot* as defined by Equation (9). **(B)** Shows the HRB sub-technique with high *armMo*, *armCorr*, and ψ_*ski*_ as defined in Equation (28) illustrated by subject 3. Lastly, the noTech class, corresponding to Equation (10), where the motion estimators do not fit any defined sub-technique, is shown in **(C)** by subject 1. Subject 3 was included in this illustration since no video of subject 1 performing HRB was available. The figures are single video frames from a full video of the subjects synchronized with the sensor data and motion estimators. An example video is available in the Supplementary Materials.

Figures 5–**7** display examples of all sub-techniques defined in Equations (5) to (10) and (28), and illustrates how the decision functions from the proof of Proposition 1 can be used together with the implemented motion estimators from section 2.3 to classify the classical XC skiing sub-techniques. Note that the chest sensor data were available and depicted in the figures, but not used in the sub-technique classification. The figures include a single video frame from a full video of the subjects synchronized with the sensor data and motion estimators. An example video is available as Supplementary Materials.

The importance of the arm correlation as a motion component is clear when comparing the plots in Figures 5, **7B** with the plots in Figure 6. The arm double poling motion in DP, DK, and DPrK exhibits a correlation close to one, while the diagonal motions in DIA and HRB give correlations far below zero. This makes the arm correlation a good discriminator together with a measure of the arm motion energy, underlining Remark 3, which also discriminates the rK and noTech sub-techniques classes (see Figures 7A,C). The double poling sub-techniques (DP, DK, DPrK), shown in Figure 6, are discriminated by the leg work components, *legMoS*, *legMoST*, and *kickRot*. These reflect motion in the sagittal plane, in both sagittal and transversal planes and the rotation around the vertical axes. Other more direct angular rate discriminators, and motion components, could for these cases be implemented. However, the combined leg motion estimators showed robust and well-behaved properties even though the sensors were mounted on the skis instead of the lower legs. It is worth mentioning that with the sensor mounted on the ski, the leg motion was not directly recorded and can only be inferred by the ski motion through the spring-damper hinge connection (binding) between the foot and the ski.

The DIA and HRB sub-techniques are mainly separated by ψ_*Ski*_, which in the presented plots is above the threshold only for the HRB case (Figure 7B). The arm correlation threshold was set slightly higher for the HRB compared to the DIA case, see Equations (27–29), since less anti-synchronized movement was expected in this sub-technique.

In the noTech example, Figure 7C, the athlete is tucking and the **relative** motion components *kickRot* and *armCorr* have significant fluctuations above the parameters threshold, but the **absolute** measures *armMo* and *legMoST* are well below the specified thresholds. This shows that even though **absolute** motion components may be highly dependent on the athlete, the session intensity and sensor placement, it is necessary to include such components to control the signal to noise errors for the **relative** motion components and promote algorithm robustness.

The reason for the large differences between *tol*_*armMo*_, *tol*_*legMoS*_, and *tol*_*legMoST*_ is the sensor placement. The arm sensors were placed directly on the arms while the leg sensors were placed on the skis. Since the skis are fixed to the foot through bindings, the angular rate around the lateral axis was significantly less than it would have been if the sensors were placed directly on the legs. The ski to leg mapping threshold is not linear and will depend on athlete sub-technique, equipment and conditions. A more general leg motion estimator, with reference to section 2.3.2, incorporating both gyro and acceleration channels may therefore be more robust and invariant to the lower leg or ski placement. This will be considered in further work.

Skating XC skiing sub-techniques and motion cycles without pole usage are not commonly considered part of the classical XC skiing sub-technique domain (Remark 4). Exceptions are the rotational kick classes which are allowed in turns and when direction changes are necessary. rK will for example also be active when the athlete performs G5, skating without poles, and DPrK will be active during G3, the double dance sub-technique. By including more motion components, further distinctions between sub-technique classes may be defined, and a relaxation of Assumption 1 may be considered. Examples are estimators for the ski-glide/slide motion component, based on acceleration measures, discriminating between HRB and the sliding HRB, also called the diagonal skate sub-technique (G1). Coupling rotational kicks from both legs during a double poling motion will indicate G2 or G4, the paddling or single dance sub-techniques, where further discrimination will require estimates of kinematic timing and similarity of the arm work. Including more motion components may also be aimed at making the classification more precise and robust. The correlation between the angular rate of the arms and legs may for example be used in the DIA definition, but also discriminate the amble gait as suggested in Remark 1.

By introducing other sensor systems like magnetometers, which are common in many IMU packages, ski absolute orientation may be estimated through the electromagnetic field of the earth. The drawback of using this measure directly is due to the local variations of the field. However, the relative orientation between the skis may be robustly identified under the assumption that the skis are in the same but distorted electromagnetic field. Magnetometer information was unfortunately not available for this work, but will be considered in further studies.

The methodology in this work promotes the understanding of the underlying motion mechanisms gained from motion sensors placed on the athlete's extremities. The derived motion components and associated thresholds are used directly in the definition of the sub-technique decision functions. This gives a basis for converging to common definitions by agreeing on the structure, the motion components, and the threshold values for discriminating the sub-technique definitions. Setting the threshold for ski orientation at the point where the DIA sub-technique shifts to HRB, or the threshold for kick rotation or direction to where the DK turns to DPrK, should therefore be a quest for precise and commonly accepted definitions. The parameters can be agreed upon through open discussions in the community or less directly found through an identification process, similar to what would be the case for algorithm calibration, based on labeled data provided by the different parties.

### 4.3. Algorithm Results Compared With Domain Expert Labels

**Figure 8:**
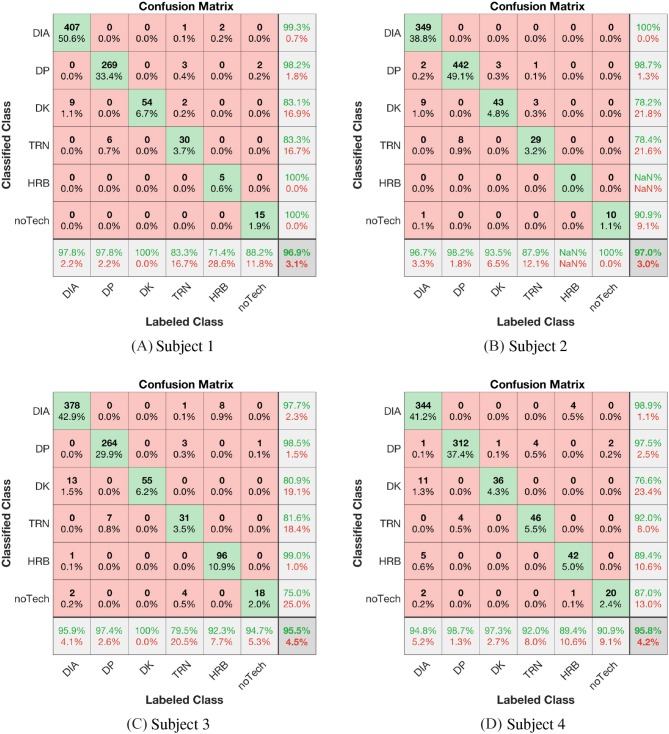
**(A–D)** Confusion matrices for subjects 1 to 4 with the mapping of the labeled sub techniques fDIA and tDIA to DIA, thus using mapping a) in Definition 3.

**Figure 9:**
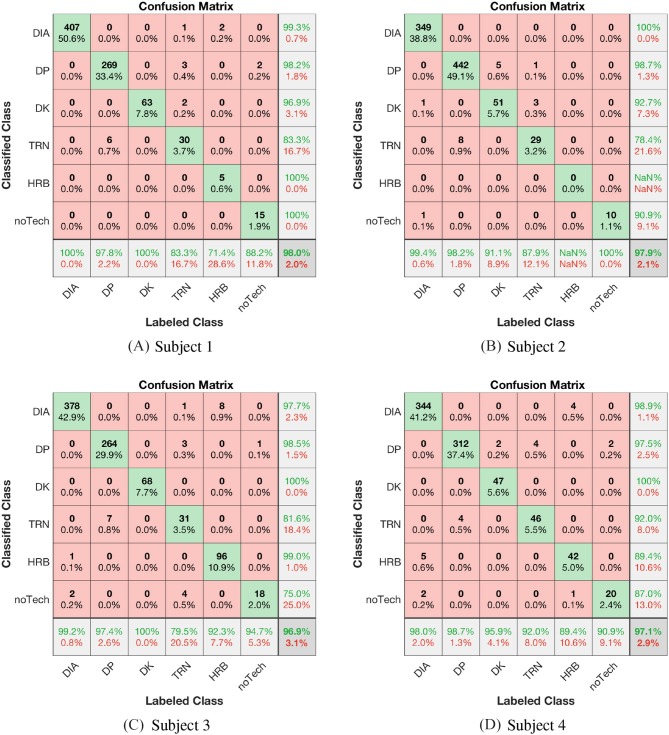
**(A–D)** Confusion matrices for subjects 1 to 4 with the mapping of the labeled sub techniques tDIA to DIA and fDIA to DK, thus using mapping b) in Definition 3.

In order to show the algorithm identification validity and discuss the challenges with the lack of quantifiable sub-technique definitions, the classification results are compared with expert-labeled data. The main tool for this comparison is the confusion matrices presented in Figures 8, 9, where the manually labeled (Labeled classes) classes are given by the columns and the algorithm labeled classes are arranged in rows (Classified classes). For the confusion matrices in Figure 8 we use the mapping **a)** of labeled sub-techniques as defined in Definition 3, thus mapping both tDIA and fDIA to DIA. The most significant label disagreement is then type II, false negative, error between algorithm DK and manual DIA labels. See for example Figure 8C where 13 cycles were classified as DK, but the expert labeled as DIA. This may seem to be a strange disagreement as the arm correlation is a strong and generally robust discriminant. However, the reason for this disagreement lies within the Definitions 3 and that these cycles are in the transition from DIA to typically DK or DP. These cycles, or half cycles (as they often are shorter), contain mainly correlated arm movement and leg work and are thus in accordance with the presented decision functions classified as DK, which is not in accordance with the fDIA rule in Definition 3 **a)** where these cycles were considered an extension of the DIA class. This is an example at the core motivation for the proposed framework, highlighting the discussion of how new expert-classified sub-technique classes are to be interpreted. In this particular case, the transition techniques are not implemented, but it shows which of the defined motion components are most significant within the cycle. This gives input to the mapping rules in Definition 3, but also suggests that the sub-technique mechanisms need to be better understood such that common and measurable definitions may be derived. However, if we use the mapping rule **b)** in Definition 3, thus mapping the labeled tDIA to DIA and fDIA to DK, we get the confusion matrices shown in Figure 9. If we compare the confusion matrices in Figures 8, 9 we observe that changing the label improves the precision in the rightmost column for the DK class from 83.1%, 78.2%, 80.9, and 76% for subjects 1, 2, 3, and 4, respectively, to 96.9%, 92.7%, 100, and 100%. Thus, the achieved precision for DK and the overall classification accuracy improve significantly. However, some XC experts might disagree on whether a DK cycle occurs in the transition between DIA and DP even though the algorithm predicts a DK cycle. On the other hand, knowing the definition of a DK cycle from Equation (7), it is a correct prediction—but such single cycles can also simply be filtered out in a post-processing of the classified cycles to agree with an XC expert's opinion. Or, one might argue that the XC expert is wrong, and that more detailed sub-technique definitions and classification algorithms can bring new knowledge into the field.

The comparison of HRB vs. DIA also led to type I and II disagreement, in which the reasons are two-fold. First, the necessary HRB motion components were not directly available from the sensor system, i.e., a model was developed relying on the athlete's usage of the ski edges during HRB. It was however observed that some athletes edged the skis, seemingly to produce more friction, when climbing a slope in DIA. The second reason is related to the tolerance definitions and specifying the boundary between HRB and DIA legwork (parallel vs. angled skis). The remaining disagreements found in the matrices are also related to tolerances of the arm and leg work in general, i.e., defining the tolerance for leg work between DP, DK, and DPrK (included in the TRN label). In the cases where arm work is not correlated enough to be considered double poling type sub-techniques, nor anti-correlated enough to be considered diagonal type sub-techniques. These tolerances are not systematically tuned but only manually set by inspecting the range of the sensor data. Proper definitions of the sub-techniques require these parameters to be set in an unbiased manner through standardization work involving XC skiing community experts.

The accuracies for the four labeled subjects can be read from the lower right in the confusion matrices in Figure 9. A total of 11 cycles were left out of the four data sets, which consists of 3427 cycles, since the XC expert found those cycles to not match any of the sub-techniques. These cycles were unusual movements, such as when the skier was checking the watch on the arm. The data sets from the four subjects received accuracies of 98%, 98%, 97, and 97%, respectively. DIA, DK, and DP exhibited excellent classification accuracies, with sensitivity and precision mostly approaching 100, whereas the TRN, HRB, and noTech classes exhibited somewhat lower sensitivity and precision due to lack of quantitative definitions and indirect motion component measures.

### 4.4. Framework Discussion

The presented approach is based on describing the mechanisms of classical XC skiing. It is expert and data driven in the sense that the models are derived based on the known kinematics of the sport, as well as the placement and types of sensors used for the motion components. When placing IMU sensors on the body extremities, the data produced is closely related to a video stream in the sense that the angular rates reflect a course estimate of the athlete kinematics. This is advantageous as it will provide a relatively direct and simple mapping, with the decision function description, to manual video analysis labeling for common qualitative sub-technique definitions. This is shown in Figures 5–7, where the motion component time series are synchronized with the video frames.

The motion components and decision functions from Definition 1 and the proof of Proposition 1 are used for classification under activity constraints. This means that the results are only valid in the case when the technique style, i.e., classical XC skiing, is known beforehand, see Assumption 1 and Remark 1. A relaxation of these constraints can be considered by extending the motion component set, adding decision functions that describe other definable sub-techniques, for example including the skating XC skiing style, as is also discussed in section 4.2. The framework does not propose a concrete recipe for composing the motion components and decision functions, meaning that different constructions may be considered, as highlighted in Remarks 2 and 3. As an example, different sensor systems can be applied as long as the derived motion components are unique and the decision functions map to an established sub-technique definition.

A main contribution from this paper is to provide a common communication platform for researchers in this field. In addition, clarifying the basic motion components and identifying sub-techniques are of high relevance for coaches and athletes in the development and utilization of digital coaching tools. For example, the Norwegian XC skiing team has systematically used technological tools combining GPS and IMUs to analyze where and why skiers gain or lose time during training and competitions as part of their preparations for major championships in the last years. In this context, detection of sub-techniques and their temporal patterns provides understanding of performance differences related to different pacing strategies, it allows detection of possible effects of training or technique changes and it may help in the optimization of tactical choices in a given track. More detailed understanding of these aspects can provide important decision support both in training and in competition settings, thereby being important coaching tools and parts of the mental strategies when preparing for competitions.

Four IMU sensors, placed on arms and legs, are used to exemplify the methodology presented in this paper. However, pose tracking video analysis or information from marker-based camera systems may also provide sufficient information for estimating the proposed motion components. In cases where a single IMU sensor is used, for example when placed on the sternum, the proposed motion components cannot be estimated directly. However, comparable results can be achieved by building a model, based upon recorded data annotated/labeled in accordance with the sub-technique definitions, motion components and decision functions. In such a case, the motion components will be implicitly represented in the trained model. However, care should be taken when applying a single sensor setup for classification of sub-technique definitions that are based on properties from the movement of more than one segment, as these properties may not in general be observable through the data provided by the one sensor.

In order to perform a proper validation of this implementation, a group of experts representing the main stakeholders in the XC skiing community is required to label the same data sets individually. This may produce data that are less biased and give insight into what the common definitions of the sub-techniques should be, including the formal definitions of the decision functions; the motion components and the constraining tolerances (see Remark 4). The presented approach has similarities to research within the physiotherapy and rehabilitation domain. In order to collect valid, reliable and comparable data on health and disability at both individual and population levels, the WHO has established The International Classification of Functioning, Disability and Health (ICF) standard (World Healt Organization, 2001). Langhammer and Stanghelle (2011) suggests that the Movement Quality Model (MQM) from the work by Skjaerven et al. (2008) relates to *body structures* and *body functions* in the ICF concept. The MQM represented the essential features and characteristics of the movement quality, reflecting a group of physiotherapists. These concepts are analogous to the framework presented in this work and are in line with the suggested need of gathering a group of XC skiing experts to label and formulate motion components to establish more detailed quantifiable sub-technique descriptions.

The concept may also be taken further in large-scale heterogeneous motion data sets of XC skiing, and sport activities in general, including raw data of video, positioning, and motion information annotated with for example athlete context, cycles, sub-techniques, and motion components. Such openly available data sets may have great impact on research and application development and would be comparable to for example the Common Objects in Context (COCO) project (Lin et al., 2014), which is very successful within the computer vision and pattern recognition domain. 2D video-based pose tracking/estimation have in recent years had great progress, extending image segmentation methods and providing absolute information for human kinematics—see the PoseTrack large-scale benchmark data set (Andriluka et al., 2018) and the DensePose project (Alp Güler et al., 2018). Including pose information as context in a heterogeneous motion data set would also provide absolute information for auto-calibrating of distributed motion sensors and validation of case study protocols. Such data sets and accompanying infrastructure may be hosted open source by the university or institute sector generally for research, or by for example FIS as a basis for building automatic classification decision support in competition regulation.

## 5. Conclusion

This work proposes a framework for building quantitative measurable definitions of the sub-techniques in classical XC skiing. It relies on a definition of motion components and uses the current qualitative definitions of the sub-techniques to produce quantitative decision functions that uniquely map an athlete's motion to each sub-technique class. The structure of this identification process is closely related to the manual video analysis technique annotation given by an expert, as it focuses on measuring and describing the motion and mechanisms in the kinematic patterns of the athlete. The approach is generic and relies on known and understood mechanisms within an activity. On a structural level it also bears similarity to the notion “quality of movement” used both in sports and in the rehabilitation domain, especially in cases where the movement can be considered cyclical. In our specific approaches, the most common sub-technique decision functions (DIA, DK, and DP) were first proposed in Seeberg et al. (2017) and thereafter extended in Solli et al. (2018) to include HRB and TRN techniques. Solli et al. (2018) also present an example on how this detailed sub-technique identification may be used to test new research hypotheses in the field. Here, a holistic presentation of the methodology, including the estimators, is given. In addition, TRN is further refined to DPrK and rK. Finally the framework implementation used in our study is compared with high“fit scores” to a data set independently labeled by a domain expert. However, commonly accepted utilization of automatic tools for sub-technique classification will require work toward more unified and quantifiable definitions of the specific sub-techniques, which has also been the overarching aim of usage for the proposed framework and methodology. Altogether, this understanding contributes to the field by providing a common communication platform of high relevance for researchers in the field, and for the further development of preparation and debriefing tools where combined GPS and IMUs help XC skiing coaches and athletes in their decision-making in training and competitions.

## Data Availability

The datasets for this manuscript are not publicly available, but all data relevant for the purpose and conclusions of this study is provided in the article. Requests to access the datasets should be directed to oyvind.sandbakk@ntnu.no.

## Ethics Statement

The study was pre-approved by the Norwegian Centre for Research Data, and conducted in accordance with the Declaration of Helsinki assured by the responsible institution, Norwegian University of Science and Technology. All participants were fully informed of all test protocols and procedures before they provided their written consent to participate.

## Author Contributions

JT wrote the paper, implemented the algorithms and created figures. TS designed the sensor system and modified the sensors to fit the purpose of this study, and contributed to conducting the field trials and writing the paper. OR contributed to writing the paper, the creation of figures, in algorithm comparison to manual labeling, and by conducting field trials. PH contributed to writing the paper, in the creation of figures, and by conducting field trials and labeling the data sets. ØS provided expert knowledge of the field, and contributed to designing the experiments and writing the paper.

### Conflict of Interest Statement

The authors declare that the research was conducted in the absence of any commercial or financial relationships that could be construed as a potential conflict of interest.

## Footnotes

^1^https://res.cloudinary.com/fis-production/image/upload/v1540201631/fis-prod/ICR_Cross-Country_2018_clean.pdf

^2^www.fis-ski.com
